# Multifunctional tendon-mimetic hydrogels

**DOI:** 10.1126/sciadv.ade6973

**Published:** 2023-02-17

**Authors:** Mingze Sun, Hegeng Li, Yong Hou, Nan Huang, Xingyu Xia, Hengjia Zhu, Qin Xu, Yuan Lin, Lizhi Xu

**Affiliations:** ^1^ Department of Mechanical Engineering, The University of Hong Kong, Hong Kong SAR, China.; ^2^ Department of Electrical and Electronic Engineering, The University of Hong Kong, Hong Kong SAR, China.; ^3^ Department of Physics, Faculty of Sciences, The Hong Kong University of Science and Technology, Hong Kong SAR, China.; ^4^ Advanced Biomedical Instrumentation Centre Limited, Hong Kong SAR, China.

## Abstract

We report multifunctional tendon-mimetic hydrogels constructed from anisotropic assembly of aramid nanofiber composites. The stiff nanofibers and soft polyvinyl alcohol in these anisotropic composite hydrogels (ACHs) mimic the structural interplay between aligned collagen fibers and proteoglycans in tendons. The ACHs exhibit a high modulus of ~1.1 GPa, strength of ~72 MPa, fracture toughness of 7333 J/m^2^, and many additional characteristics matching those of natural tendons, which was not achieved with previous synthetic hydrogels. The surfaces of ACHs were functionalized with bioactive molecules to present biophysical cues for the modulation of morphology, phenotypes, and other behaviors of attached cells. Moreover, soft bioelectronic components can be integrated on ACHs, enabling in situ sensing of various physiological parameters. The outstanding mechanics and functionality of these tendon mimetics suggest their further applications in advanced tissue engineering, implantable prosthetics, human-machine interactions, and other technologies.

## Teaser

Anisotropic assembly of nanofiber composites were exploited to construct tendon-mimetic hydrogels.

## INTRODUCTION

Reconciling the mismatches between natural biological tissues and engineering materials represents a critical demand for the development of advanced biomedical devices and tissue engineering platforms (*1*, *2*). However, natural tissues exhibit many characteristics that are difficult to replicate with synthetic materials. For instance, tendons involve hierarchical organization of aligned collagen fibers interlaced with soft water-retaining biopolymers. They contain ~60 weight % (wt %) of water while exhibiting high moduli at the gigapascal level and strengths in the range of 55 to 120 MPa (*3*, *4*). The anisotropic structures of tendons not only enable essential load-bearing capabilities for the musculoskeletal system but also provide important biophysical cues that translate into the behaviors of cells through interfacial interactions (*5*).

Over the past decade, extensive research efforts were devoted to the engineering of tendon-mimetic materials with high structural anisotropy. For instance, tensile stress was exploited for the orientation of polymer networks, leading to hydrogels with enhanced mechanical strength along the stretched direction (*6*–*9*). Multiple networks or physical cross-linking were incorporated into hydrogels for the improvement of fracture toughness (*10*–*12*). Phase separation induced by freezing and salting out was recently explored to generate hierarchical structures, further improving the mechanics of hydrogels (*13*). However, the moduli of these anisotropic hydrogels are still orders of magnitude lower than that of the natural tendon, partly due to the flexibility of hydrophilic polymer chains in the presence of water. Incorporating bundled fibers from cellulose (*14*, *15*) or synthetic polymers (*16*, *17*) may confer high stiffness to the hydrogel composites. However, it is challenging to control the interactions between the stiff fibers and soft matrix to emulate the microstructural interplay in load-bearing soft tissues. Therefore, many mechanical behaviors of the natural tendons, such as strain-stiffening and viscoelastic responses, remain difficult to replicate with fiber-reinforced hydrogels. Furthermore, previous efforts on tendon-mimetic materials mostly focused on the engineering of mechanical properties. Limited attention was paid to the functionalization of materials that enables bioactive interfaces with cells and tissues, therefore limiting their potential for biomedical applications.

Here, we report a materials platform for the construction of hybrid anisotropic hydrogels with tendon-like behaviors and multifunctionality for biointerfaces. Reconfigurable interactions between stiff aramid nanofibers (ANFs) and flexible polyvinyl alcohol (PVA) allow assembly of highly oriented network that emulates the microstructural interplay between aligned collagen fibers and soft proteoglycans. The resulting anisotropic composite hydrogels (ACHs) exhibit high mechanical properties matching those of the natural tendons while retaining a similar water content of ~60%. Biofunctionalization of ACHs was made possible, providing anisotropic biophysical cues for the modulation of cell behaviors. Soft bioelectronics can be further integrated on ACHs, enabling hybrid devices capable of in situ sensing and stimulation. The mechanics and functionality of these tendon mimetics indicate opportunities for their application in advanced biomedical technologies.

## RESULTS

**Fig. 1.**
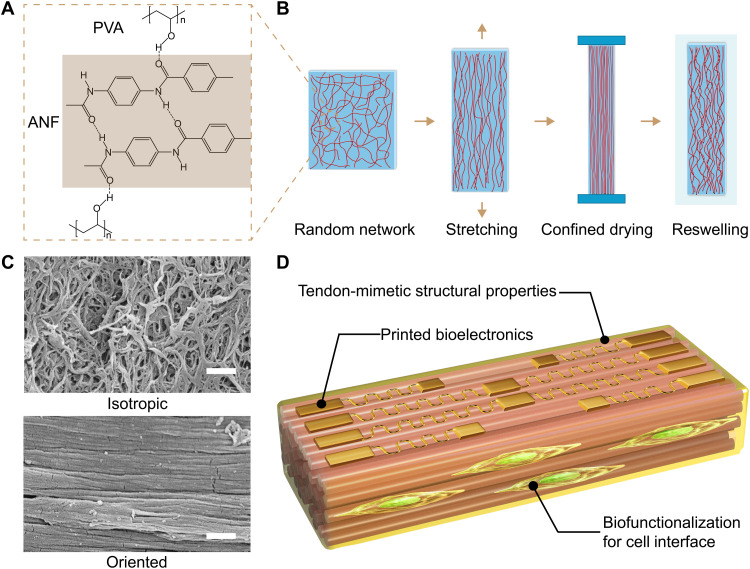
Design and processing of tendon-mimetic ACHs (**A**) Chemical structures of ANF and PVA and their intermolecular hydrogen bonding. (**B**) Schematics of the processing steps for ACH involving stretching and confined drying for the orientation of nanofiber assembly. (**C**) SEM images of isotropic ANF-PVA hydrogel (top) and ACH-80 (bottom). Scale bars, 1 μm. (**D**) Schematics of the multifunctional tendon-mimetic ACHs.

Fabrication of ACH involves stretching and confined drying applied to isotropic hydrogels consisting of ANFs and PVA. ANFs exhibit branched microstructures with fiber diameters of 5 to 30 nm and lengths of 3 to 10 μm, providing collagen-mimetic building blocks for the composites (*18*). Extensive hydrogen bonding between ANFs and PVA confers reconfigurability on the three-dimensional (3D) network combined with high toughness (Fig. 1A). Under uniaxial tension, the fibrillar network orients along the direction of stretching without structural disintegration even under 80% of strain (Fig. 1B and fig. S1). Next, the hydrogel samples underwent drying in atmosphere (~50% in humidity) with their length fixed in the direction of stretching (table S1). This step facilitates interfibrillar interactions under the confined configuration, leading to permanent alignment of the fibrillar network (*19*). Anisotropic reswelling behaviors of the samples in aqueous media resulted in ACHs with an equilibrium water content of ~60 to 74%, similar to those of natural tendons (table S1). Scanning electron microscopy (SEM) images confirmed the highly oriented fibrillar network of ACH in contrast with that of isotropic ANF-PVA hydrogels (Fig. 1C). In addition, bundling and crimping of fibers were observed in ACH, resembling the hierarchical structures in natural tendons (fig. S2). The anisotropic microstructures appeared consistent across the entire depth of millimeter-scale samples (fig. S3), leading to robust mechanical behaviors of ACHs. Moreover, the PVA chains afford further chemical functionalization, enabling bioactive interfaces for cells or integration with multifunctional bioelectronic devices (Fig. 1D).

**Fig. 2.**
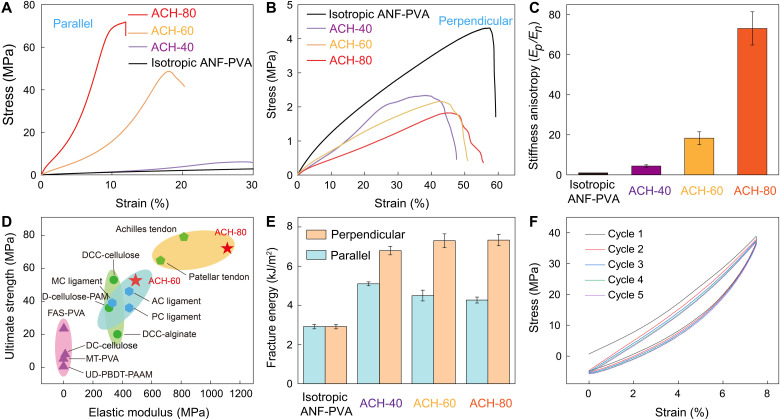
Mechanics of ACHs. (**A** and **B**) Tensile stress-strain curves of ACHs in the directions parallel (A) and perpendicular (B) to the fiber orientation, respectively, as compared with the responses of isotropic ANF-PVA hydrogels. The sample denoted as ACH-*x* corresponds to *x*% of imposed elongation during the prestretching-drying process. (**C**) Stiffness anisotropy of ACHs, as characterized by the ratio between initial tensile moduli parallel (*E_p_*) and perpendicular/normal (*E_n_*) to the fiber alignment. (**D**) Moduli and strengths of ACH-80 and ACH-60 as compared with those of natural tendons, ligaments, and other anisotropic hydrogels with tendon-mimetic characteristics (table S2). (**E**) Fracture energies of ACHs measured in the directions parallel and perpendicular to the fiber alignment, as compared with those of isotropic ANF-PVA hydrogels. (**F**) Cyclic tensile tests on ACH-80 in the direction parallel to the fiber alignment, with 7.5% of maximum imposed strain.

The stretching-induced orientation of the fibrillar networks influenced the mechanics of ACHs. We investigated various samples denoted as ACH-*x*, with *x* being the percentage of imposed elongation during the prestretching-drying process. From microstructural observations, the degree of fiber alignment and bundling in ACHs increases with elongation during the prestretching-drying processing (fig. S4). In addition, the interfibrillar interactions in highly stretching-oriented samples (e.g., ACH-80) led to a high solid content in the swollen state (~40%), which contrasts with those processed with lower prestretching (fig. S5). As a result, moduli and strengths of ACHs in the direction parallel to the fiber alignment strongly correlate with the degree of imposed elongation during prestretching drying (Fig. 2A), with ACH-80 exhibiting the highest elastic modulus of 1.1 GPa and strength of 72.1 MPa (figs. S6 and S7). These values are ~65 and ~10 times higher than the modulus and strength of isotropic ANF-PVA hydrogels with similar water content (figs. S8 and S9), indicating the contribution of microstructural anisotropy for the mechanics of ACHs. On the other hand, stretching-induced orientation led to a decrease of strength of ACHs in the direction perpendicular to the fiber alignment, partly due to the diminishing contribution of stiff fibers for load bearing (Fig. 2B). The stiffness anisotropy of ACHs, as characterized by the ratio between initial tensile moduli parallel and perpendicular to the fiber alignment, is tunable up to the level of ~80 (Fig. 2C), which covers the intrinsic range of biological tissues (*20*, *21*). Notably, the elastic modulus and mechanical strength of ACH-80 match those of the natural tendons, which were not achieved with other synthetic hydrogels with tendon-mimetic characteristics (Fig. 2D). The properties are stable in aqueous environment (fig. S10). Moreover, ACH exhibit high toughness in both directions parallel and perpendicular to the prestretching (Fig. 2E and fig. S11). Markedly, the aligned fibers in ACHs provide enhanced resistance to crack propagation in the direction perpendicular to their orientation, leading to a fracture energy of as high as 7333 J/m^2^.

Many tendon-mimetic mechanical properties of ACHs originate from the interplay among the nanoscale constituents. For instance, the reconfigurable hydrogen bonding between ANFs and PVA imparts high plasticity to the network, affording stress-induced orientation for ACHs. In addition, the 3D fibrillar network with high-strength nodes bonded by PVA provides excellent load-bearing capabilities. Markedly, ACH-80 sustained a maximum stress of as high as ~39 MPa even under cyclic elongation of 7.5%, indicating high structural robustness (Fig. 2F). The fiber crimping in ACHs could be resulted from the reconfiguration of flexible PVA chains after removing the prestretching (table S1). This feature imparts strain-stiffening behaviors of ACHs (Fig. 2, A and F). It was observed that tangent modulus of ACH-80 ranges from 553.8 to 1157.2 MPa depending on the imposed tensile strain (fig. S7). Furthermore, the abundant noncovalent intermolecular interactions in ACHs also led to viscoelastic responses, as evidenced by hysteresis under cyclic loadings (Fig. 2F and fig. S12) and stress relaxation with a time constant on the order of ~10 s (fig. S13), which are very similar to the properties of load-bearing soft tissues (*22*). These tissue-mimetic mechanical behaviors of ACHs, together, may create possibilities for the construction of advanced biointerfaces.

We next investigated whether the structural characteristics of ACHs can influence the behaviors of cells through interfacial interactions. To promote cell adhesion and mechanosensing on ACHs, we adopted chemical functionalization to present arginylglycylaspartic acid (RGD) motifs for the binding with integrins on the cell membrane. Specifically, benzophenone (BPh) functionalized amphiphilic block copolymers involving linear polyglycerol (LPG) were adsorbed on the surface of ACH in an aqueous environment. The terminal group of the hydrophilic LPG units was linked to integrin-binding motif cycloRGDfK (*23*). Under illumination with ultraviolet (UV) light, the BPh groups grafted to the backbone of PVA and cross-linked with adjacent block copolymers via hydrogen atom abstraction, leading to a functional coating covalently bonded with ACHs (fig. S14). Successful biofunctionalization for ACHs was evidenced by the adhesion of NIH3T3 fibroblasts on their surfaces showing typical spindle-like morphology (fig. S14). In contrast, samples without surface functionalization led to little attachment of cells. The good cytocompatibility of ACHs was also confirmed with MTT [3-(4,5-dimethylthiazol-2-yl)-2,5-diphenyltetrazolium bromide] assay (fig. S15).

**Fig. 3.**
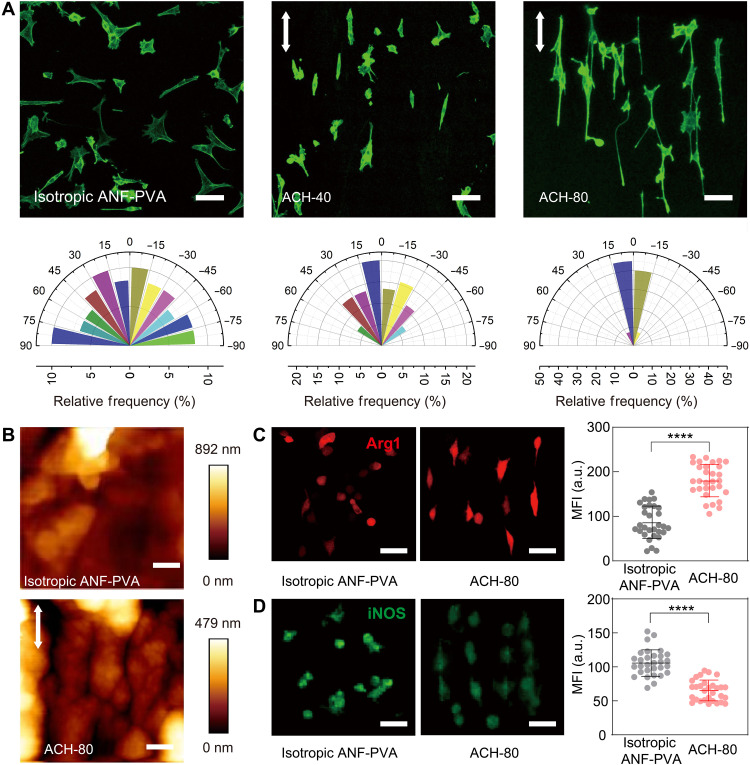
Regulating cell morphology and phenotypes with biofunctionalized ACHs. (**A**) Fluorescence images of F-actin in NIH-3T3 fibroblasts cultured on various substrates (top) and the corresponding angular distribution of cell orientation (bottom) (*n* ≥ 30). Zero angle (0°) represents the direction parallel to the fiber alignment. Scale bars, 100 μm. (**B**) AFM images showing the surface topography of biofunctionalized ACH-80 (bottom) and isotropic ANF-PVA hydrogel (top). Scale bars, 1 μm. (**C**) Fluorescence images of RAW 264.7 macrophages cultured on isotropic ANF-PVA hydrogel (left) and ACH-80 (middle), immunostained for M2 biomarker Arg1, and statistics of the mean fluorescence intensity (MFI) of individual cells showing the differences induced by distinct substrates (right). The cell cultures were treated with IL-4 and IL-13 to induce M2 phenotype. Scale bars, 50 μm. a.u., arbitrary units(**D**) Immunostaining for iNOS (M1 biomarker) in RAW 264.7 showing the distinct effects induced by isotropic ANF-PVA (left) and ACH-80 (right), also characterized by MFI statistics (right). IFN-γ and LPS were used to induce M1 phenotype. Scale bars, 50 μm. *n* = 30, ****P < 0.0001. All white arrows indicate the direction parallel to the fiber alignment.

The structural anisotropy of ACHs clearly translated into the morphology of attached cells. While isotropic ANF-PVA hydrogels led to random arrangement of the attached fibroblasts, ACHs induced significant orientation of cells, with the degree of orientation increasing with the alignment of fibers (Fig. 3A). Morphology of cells could be regulated by various structural factors of the extracellular matrix, such anisotropy of stiffness (*24*), surface topography (*25*), or viscoelastic responses (*26*). ACHs present many of these characteristics, which could collectively influence the behaviors of attached cells through mechanosensing and mechanotransduction. Further examinations indicated that self-assembled surface topography is the major contributor for the cell orientation on ACHs. Specifically, both atomic force microscopy (AFM) (Fig. 3B) and optical microscopy (fig. S16) showed aligned microgrooves on the surface of ACHs with pitch sizes ranging from 0.2 to 20 μm, which provide contact guidance for cell orientation (*25*). On the other hand, removal of the surface topography on ACH with plasma etching led to much less orientation of the attached cells (fig. S17). It is known that the contractile machinery of cells mediated by Rho-associated protein kinase (ROCK) plays an important role in their morphological responses to surface topography and substrate mechanics (*27*). For the fibroblasts cultured on ACHs, adding ROCK inhibitor Y-27623 led to diminished orientation of the cells (fig. S18), which confirmed the mechanosensing and mechanotransduction processes at the cell-ACH interfaces for the anisotropic morphogenesis.

Capabilities in regulating macrophage polarization between proinflammatory M1 and prohealing M2 phenotypes are highly desirable for tissue engineering constructs (*28*, *29*). Recent studies showed that cell elongation of macrophages due to mechanotransduction can promote their polarization toward M2 phenotype (*30*). However, means for controlling macrophage behaviors were rarely demonstrated on a tendon-mimetic materials platform. We next investigated whether the anisotropic structural features of ACHs could influence the morphology of attached macrophages and regulate their polarization. Notably, RAW 264.7 macrophages cultured on ACH-80 exhibit significant orientation and elongation in accordance with the substrate anisotropy, which contrasts with those cultured on isotropic ANF-PVA hydrogels (Fig. 3, C and D, and fig. S19). The elongation of macrophages enhanced the effect of M2-inducing cytokines interleukin-4 (IL-4) and IL-13 added to the cell culture, leading to a higher expression of M2 biomarker arginase 1 (Arg1) as compared with those cultured on isotropic substrates (Fig. 3C). In another experiment, the cell elongation on ACHs counteracted with the applied M1-inducing stimuli interferon-γ (IFN-γ) and lipopolysaccharide (LPS), leading to a lower expression of inducible nitric oxide synthase (iNOS), a biomarker for M1 (Fig. 3D). These results indicate the capabilities of ACHs in promoting M2 phenotype and inhibiting M1 phenotype for macrophages, which are meaningful for further applications in implantable devices.

**Fig. 4.**
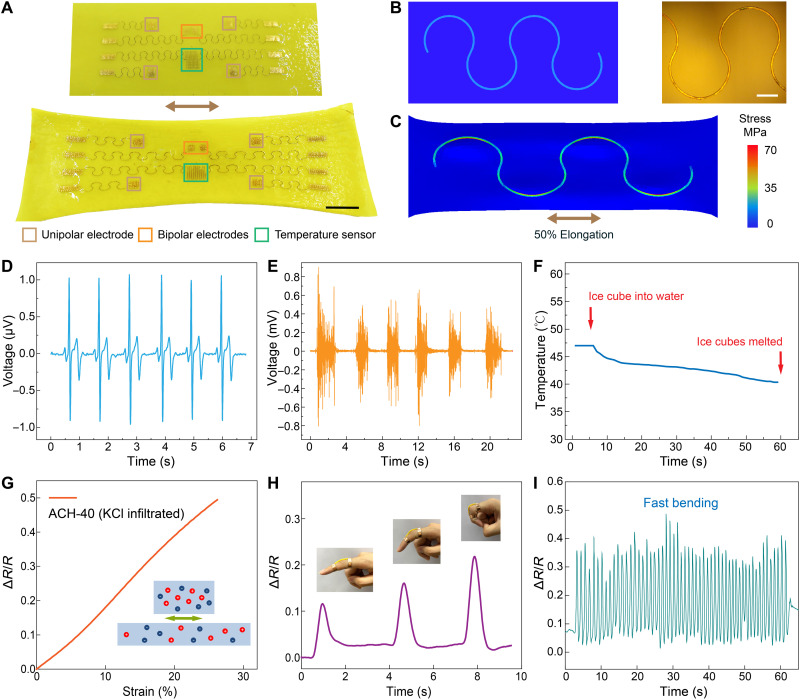
ACHs with integrated multifunctional bioelectronics. (**A**) Photographs of serpentine electronics transfer-printed onto an isotropic ANF-PVA hydrogel (top) and their stretched state with the processed ACH (bottom). The insets show various functional components. Scale bar, 2 cm. (**B**) FEA model (left) and microscope image (right) of a representative serpentine device bonded with isotropic ANF-PVA hydrogel. Scale bars, 1 mm. (**C**) FEA simulation on the stress distribution in the serpentine device under 50% elongation imposed to the hybrid structure. (**D** and **E**) ECG (D) and EMG (E) measured with bioelectrodes on a hybrid ACH. (**F**) Temperature variation in a water bath characterized with a temperature sensor on a hybrid ACH. (**G**) Schematics and the resistance response to tensile strain for an ionically conductive ACH sample. (**H** and **I**) Responses of an ACH-based strain sensor mounted on a finger under various amplitudes of deformation (H) and cyclic motion (I).

Last, we demonstrate multimodal physiological sensing with ACH via integrated soft bioelectronics. Specifically, hydroxyl groups on PVA chains in the liquid precursor of ACHs can interact with functionalized surfaces of microfabricated devices (fig. S20). After solidification by solvent exchange, a stable bonding formed between the ANF-PVA hydrogel and transfer-printed electronic components (Fig. 4A). Serpentine design was adopted for the wafer-fabricated electronics, which imparts high stretchability to withstand the prestretching-drying processing for ACHs (figs. S21 to S23). As indicated by finite element analysis (FEA), the stress distributed in a representative serpentine device during 50% of stretching is significantly below the failure threshold of the constituent materials [e.g., polyimide (PI) and gold] (Fig. 4, B and C). It is conceivable that the stretchability of the electronic component can be further enhanced by modifying their geometrical designs (*31*), which can help to retain their mechanical integrity during higher elongation. To demonstrate sensory functions, an array of bioelectrodes on ACHs was used to characterize electrophysiological signals from the skin, leading to high-quality recordings of electrocardiogram (ECG) and electromyogram (EMG) (Fig. 4, D and E). Temperature sensors based on encapsulated thin-film resistors exhibited excellent responses even in aqueous media (Fig. 4F and fig. S24), which suggests their capabilities in an implanted configuration. These functional components on ACHs are only representative. Many other biosensors and actuators could be incorporated into this platform due to the versatility of microfabrication and transfer-printing techniques (*32*). Furthermore, we engineered a strain sensor based on ionic conductivity of ACHs. Specifically, the electrical resistance of an ACH sample infiltrated with potassium chloride increases with imposed elongation, showing a typical gauge factor of ~2.5 (Fig. 4G). This ACH-based sensor is capable of characterizing motion of a joint under various amplitudes (Fig. 4H) and frequencies (Fig. 4I).

## DISCUSSION

In conclusion, we have developed tendon-mimetic hydrogels with outstanding mechanics and functionality originating from the anisotropic assembly of nanofiber composites. The biophysical cues presented by ACHs can be further used for the control of differentiation, migration, and other activities of cells, which helps to expand the toolbox for advanced tissue engineering. On the other hand, the set of tendon-mimetic behaviors of ACHs suggests their potential applications as implantable tissue prosthetics. In this regard, physical integration between ACHs and natural tissues in vivo may require further engineering attention. Multifunctional bioelectronics integrated on ACHs could provide critical capabilities for in situ monitoring of various physiological parameters. Incorporation of wireless modules (*33*) should enable fully implanted system that allow two-way communications between external control hardware and the electronically active prosthesis.

## MATERIALS AND METHODS

### Fabrication of ACHs

Three grams of Kevlar para-aramid pulp and 3.0 g of KOH were first mixed in 100 ml of dimethyl sulfoxide (DMSO), and then the mixture was magnetically stirred for 7 days at 95°C to obtain 3 wt % ANF dispersion. PVA (15.0 g; 99%+, molecular weight: 146,000 to 186,000, Sigma-Aldrich) was dissolved in 100 ml of DMSO and magnetically stirred for 3 days to obtain 15 wt % PVA solution. ANF dispersion in DMSO and PVA solution in DMSO were mixed with 1:1 mass ratio for the liquids and then casted in molds and immersed in deionized (DI) water for more than 24 hours to obtain ANF-PVA hydrogels. Isotropic ANF-PVA hydrogels were stretched with various elongation levels and dried in atmosphere for 20 hours, with the length in the stretching direction fixed. After drying, the samples were released from the loading and reswelled in DI water for 24 hours. Water/solid contents of samples were determined by the weight differences between hydrogels and their fully dehydrated states after baking in a 100°C vacuum oven for 24 hours.

### SEM and AFM characterization

A scanning electron microscope (Hitachi S4800 FEG) was used to observe the surfaces and cross sections of the hydrogels. The samples for SEM examination were prepared by a solvent exchange in ethanol and critical point drying (Tousimis Autosamdri 931). The hydrogel samples were frozen in liquid nitrogen and mechanically fractured to expose the cross-section and longitudinal section for examination. AFM (Bruker Nanowizard4 XP) was used to observe surface topography of biofunctionalized hydrogels in an aqueous environment. Silicon nitride probes (ScanAsyst-Fluid, Bruker) were used for the characterization.

### Mechanical testing

To carry out tensile tests, hydrogels were cut in a dumbbell shape and tested at room temperature with a mechanical tester (Zwick Roell) with a fixed strain rate of 100% per minute. Samples for tearing test were cut into a trouser shape, and the two arms of hydrogels were then clamped on the mechanical tester and stretched with a fixed test velocity of 1.7 mm/s. The fracture energy Γ was calculated by Γ = 2*F*/*b*, where *F* is the average steady-state tearing force and *b* is the thickness of samples. For the measurement of the stress-relaxation properties of hydrogels, the samples were stretched to various strain levels with a fixed deformation rate of 100% per minute, and the strain was retained for 60 s. The initial modulus was determined as the slope of the stress-strain curve under 2% of tensile strain.

### Surface functionalization

RGD functionalized amphiphilic block copolymer [benzophenone functionalized linear polyglycerol (LPG-BPh)] solution (1 mg/ml) was applied onto the surface of ANF-PVA hydrogels and then rested for 30 min for adsorption. Then, the coated hydrogel surface was illuminated with UV irradiation for 15 min, allowing grafting of LPG-BPh on PVA and cross-linking between LPG-BPh. The thickness of the coating is estimated as 3 nm (*34*). After rinsing with phosphate-buffered saline (PBS), the substrate is ready for cell growth.

### Fibroblast culture and immunofluorescence staining

Fibroblasts (NIH-3T3 cells) were cultured in Dulbecco’s modified Eagle’s medium with 10% fetal bovine serum and 1% penicillin/streptomycin (all from Thermo Fisher Scientific). The hydrogels were soaked in PBS (Thermo Fisher Scientific) for 24 hours and then sterilized by UV overnight. NIH-3T3 cells were seeded on the hydrogels in 24-well plates with a density of 2 × 104 cells per well and cultured for 24 hours. To evaluate the effect of ROCK, fibroblasts were cultured on hydrogels within the media containing the various concentrations of Y-27632 overnight.

The morphology of fibroblasts was observed by fluorescent staining. After culture for 24 hours, cells were fixed by 4% paraformaldehyde (Aladdin) for 15 min at room temperature and then permeabilized with 0.25% Triton X-100 (Aladdin, diluted with PBS) for 10 min and blocked by 1% bovine serum albumin (Thermo Fisher Scientific) for 1 hour. Subsequently, the cytoskeleton of fibroblasts was stained with phalloidin–iFluor 488 reagent (1:1000, diluted with PBS; Abcam) at 4°C overnight. The fibroblasts were observed with a laser scanning confocal microscope (Nikon Instruments Inc., Japan).

### Macrophage culture and polarization

Mouse macrophages (RAW 264.7) were purchased from the American Type Culture Collection. Cells were seeded on the hydrogels in 24-well plates with the density of 2 × 10^4^ cells per well and incubated for 24 hours before stimulation. Then, the incubation media were replaced by media containing IFN-γ (20 ng/ml)/LPS (100 ng/ml) or IL-4 (40 ng/ml)/IL-13 (200 ng/ml) for 24 hours.

### Macrophage immunofluorescence staining

RAW 264.7 macrophages on various hydrogels were fixed and permeabilized with the same method described above for the fibroblasts. After that, cells were further stained with iNOS monoclonal antibody (CXNFT), Alexa Fluor 488 (1:100, diluted with PBS; eBioscience, Thermo Fisher Scientific), and Arg1 monoclonal antibody (A1exF5), Alexa Fluor 488 (1:50, diluted with PBS; eBioscience, Thermo Fisher Scientific). The cells were observed with a laser scanning confocal microscope (Nikon Instruments Inc., Japan).

### Analysis of cell orientation and mean fluorescence intensity

ImageJ software was used to draw cell outline manually, which helps to determine the length of a cell’s main axis (*L*) and orientation angel (θ) (defined as the angle between the main axis of cell and the prestretching direction for ACHs). Furthermore, orientation index (*S*) was determined by the following equation: *S* = cos(2θ). The mean fluorescence intensity (MFI) of each cell was calculated from the total fluorescence intensity of a whole cell divided by the cell area. Statistics were based on measurements for at least 30 cells.

### Statistical analysis

Data were presented as mean values ± SD of at least five tests, unless otherwise indicated. An unpaired Student’s *t* test was used to evaluate the statistical significance of the variance, and *P* < 0.05 was considered statistically significant.

### Fabrication of anisotropic hydrogel integrated with electronics

Poly(methyl methacrylate) (PMMA; Sigma-Aldrich) was first spin-coated on a 4-inch silicon wafer, followed by spin coating and curing of a layer of PI (Sigma-Aldrich). Then, the top of PI layer was deposited with chromium (Cr, 5 nm) and gold (Au, 50 nm) by sputtering (Denton Desktop Pro). Photolithography was done with photoresist AZ 5214 and mask aligner URE-2000/35L. Through wet etching, the first metal layer (temperature sensors) was fabricated. Using the same method, the second metal layer (5-nm Cr and 200-nm Au) was deposited and patterned through photolithography and wet etching to obtain unipolar electrodes, bipolar electrodes, and interconnects. Another layer of PI was applied and patterned by reactive ion etching (RIE; Tailong Electronics). PMMA was completely dissolved in acetone overnight, and the microfabricated devices were picked up with water-soluble PVA tapes, leading to stamp-supported devices. To enable the robust adhesion with hydrogels, the devices were treated with RIE to create additional functional groups on the polymer surface. Then, the well-mixed ANF-PVA in DMSO was blade-coated on the top of the stamp-supported devices. After that, the whole device was submerged in DI water for releasing the tape and solidifying the ANF-PVA hydrogel. The hydrogel samples that bonded with serpentine electronics were processed with stretching, confined drying, and reswelling in DI water to obtain hybrid ACHs.

## Supplementary Materials

### This PDF file includes:

Figs. S1 to S24 Tables S1 and S2 References
