# Supplementary material for: Multifunctional tendon-mimetic hydrogels
Source: Sci Adv. 2023 Feb 17;9(7):eade6973. doi: 10.1126/sciadv.ade6973 (PMC9937573; doi:10.1126/sciadv.ade6973)
Supplement: Supplementary file 1 — Figs. S1 to S24 Tables S1 and S2 References [file sciadv.ade6973_sm.pdf]

Science Advances
AAAS

Supplementary Materials for

Multifunctional tendon-mimetic hydrogels

Mingze Sun *et al.*

Corresponding author: Lizhi Xu, xulizhi@hku.hk

Sci. Adv. **9**, eade6973 (2023)
DOI: 10.1126/sciadv.ade6973

This PDF file includes:

Figs. S1 to S24
Tables S1 and S2
References

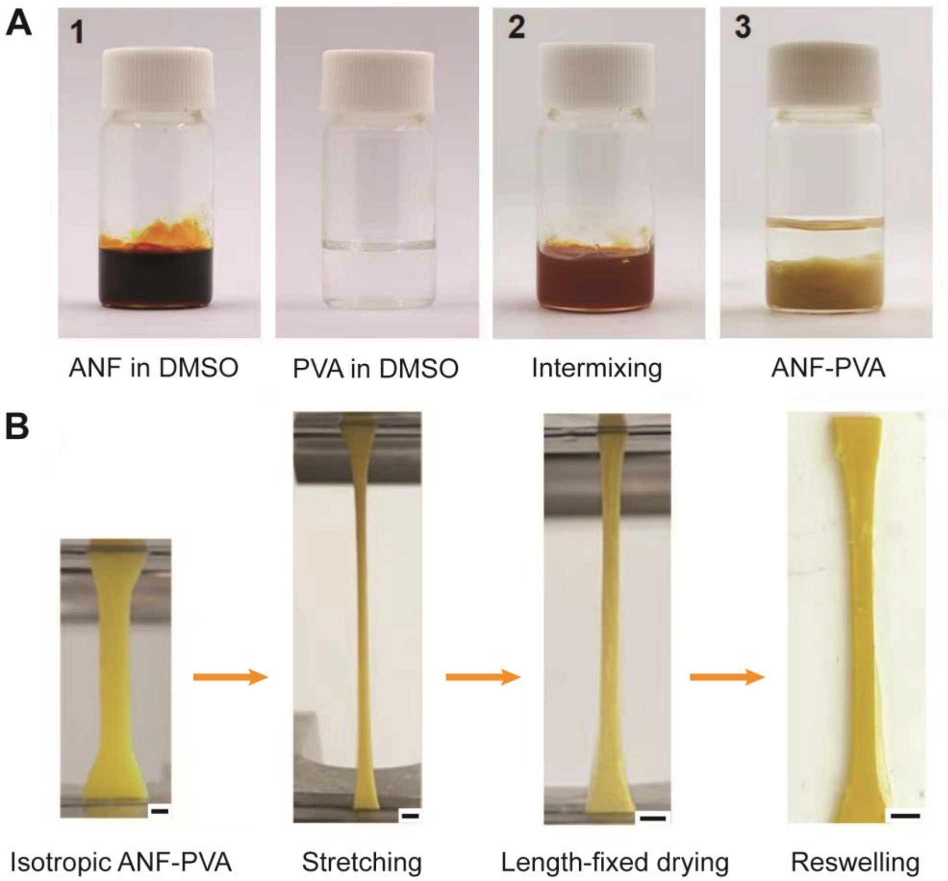

Fig. S1. Fabrication processes for ACHs. (**A**) Photographs of the materials components showing the processing for ANF-PVA hydrogels. (**B**) Photographs of an ACH during various processing steps. Scale bar: 2 mm.

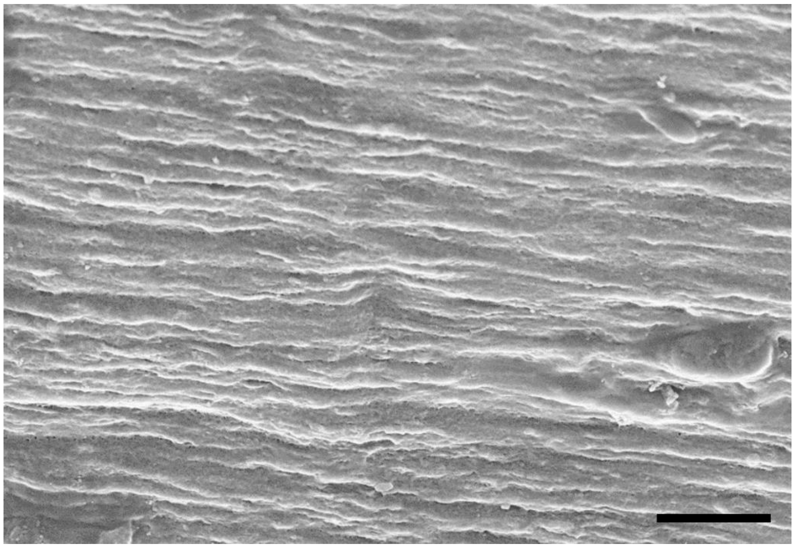

Fig. S2. A SEM image of ACH-80. Fiber bundling and crimping can be observed from the microstructural examination. Scale bar: 2 μm.

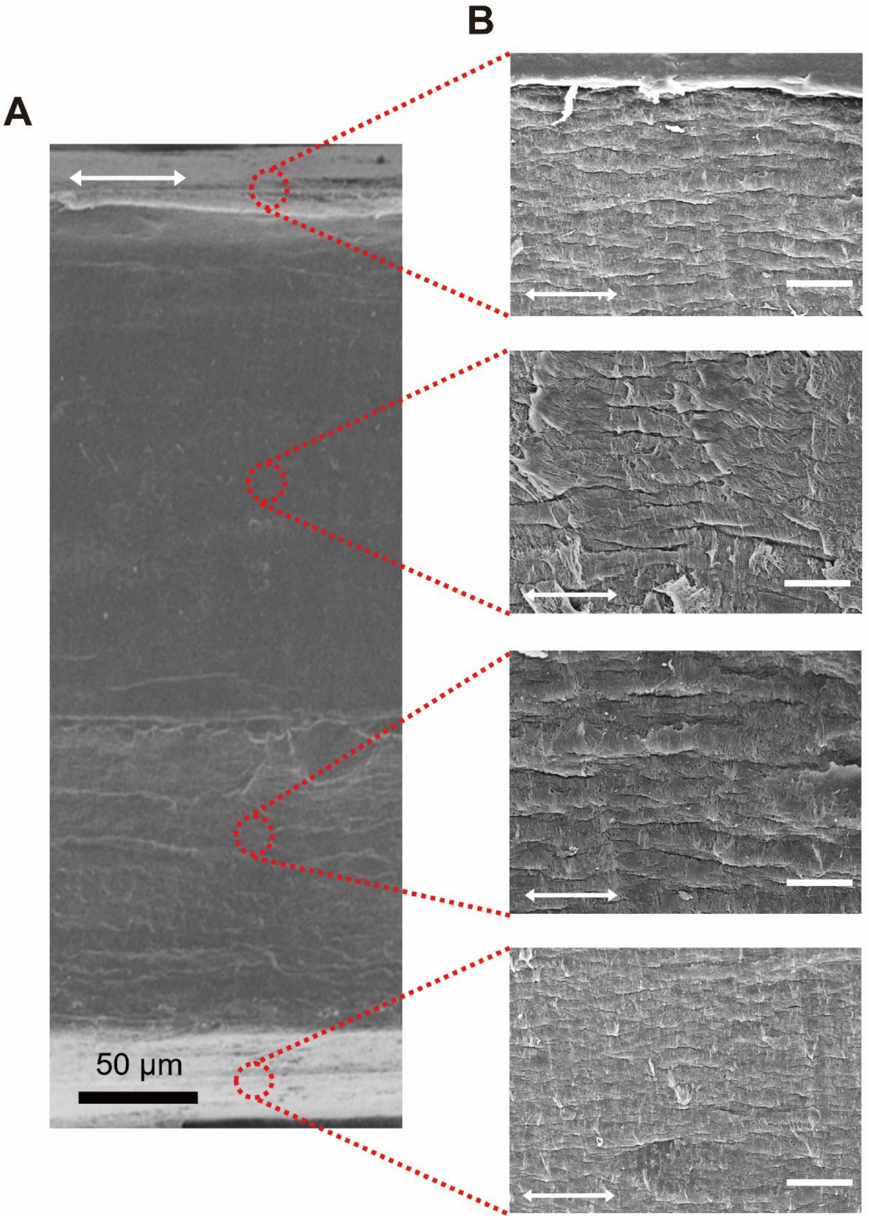

Fig. S3. SEM examination on the longitudinal section of ACH-80. (**A**) Lower magnification. (**B**) Magnified details corresponding to the layers shown in (**A**). The microstructures appeared consistent across the entire thickness of the sample. The arrows indicate the direction of pre-stretching. Scale bar: 2 μm.

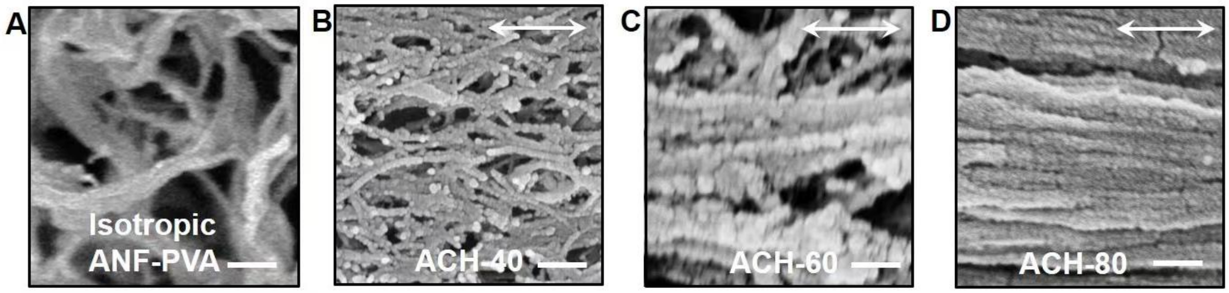

Fig. S4. SEM images of ACHs with various pre-stretching ratios (**A**) Isotropic ANF-PVA. (**B**) ACH-40. (**C**) ACH-60. (**D**) ACH-80. The degree of fiber alignment and bundling in ACHs increase with increasing elongation during the pre-stretching-drying processing. Scale bar: 100 nm.

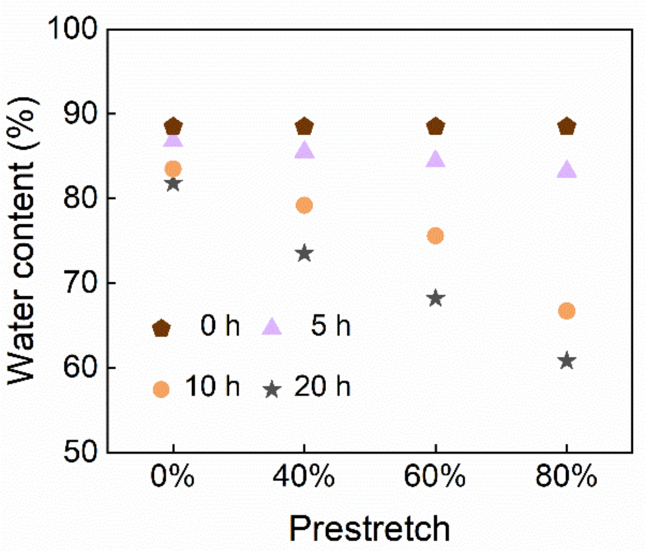

Fig. S5. Water content of ACHs as a function of pre-stretching (0 %, 40 %, 60 % and 80 %) and drying time (0 h, 5 h, 10 h, and 20 h). The samples were immersed in DI water for over 24 h to achieve equilibrium water content before the measurement.

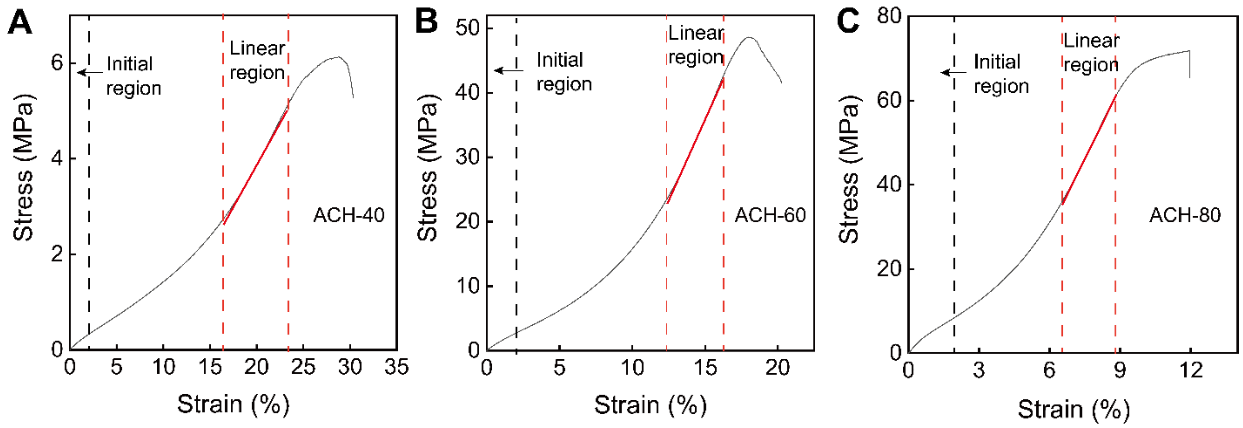

Fig. S6. Engineering stress–strain curves of ACHs highlighting different regions. (**A**) ACH-40, (**B**) ACH-60 and (**C**) ACH-80. Similar to those in natural tendons, the maximum tangent moduli occur in the "linear region", corresponding to critical load-bearing capabilities (*5*, *35*). The red line indicates linear fits for the linear regions to determine the elastic moduli of ACHs.

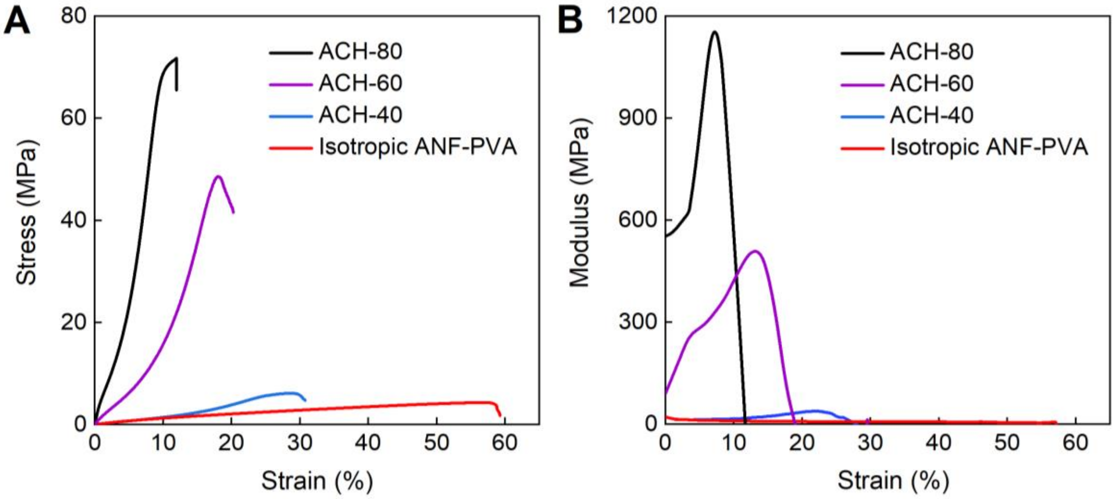

Fig. S7. Stress-strain curves and modulus-strain curves for ACHs. (A) The tensile stress-strain responses shown with full range of the applied strain until fracture of the samples. (**B)** Tangent modulus as determined by the first order derivative of the stress-strain curve. Strain-stiffening behaviors were observed in ACHs.

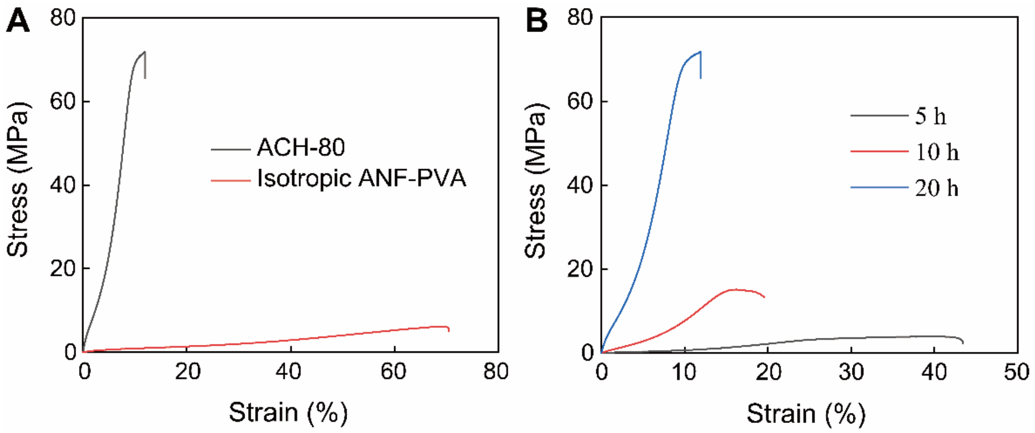

Fig. S8. Effects of pre-stretching and drying on the mechanics of ACHs. (**A**) Comparison of mechanical properties between ACH-80 and an isotropic ANF-PVA hydrogel with enhanced solid content. Both hydrogels have a water content of 60%. (**B**) Comparison of mechanical properties of 80%-stretched samples dried with various time duration (5 h, 10 h and 20 h). The samples were immersed in DI water for 24 h to achieve equilibrium water content before the measurements.

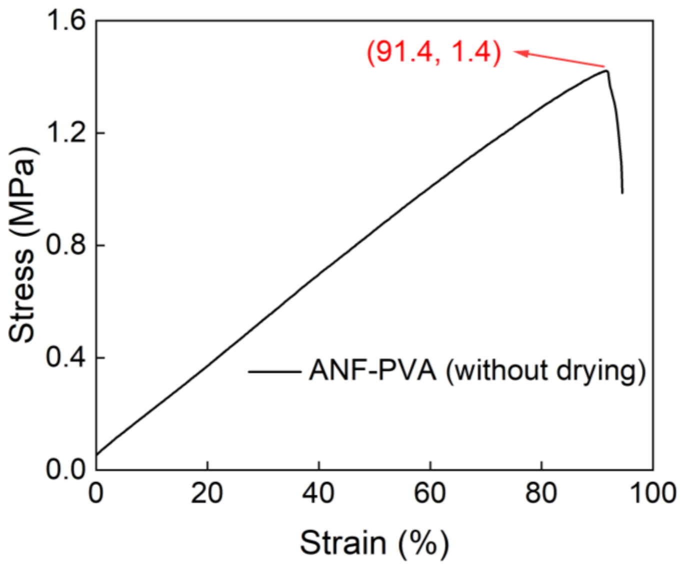

Fig. S9. Tensile behavior of the original ANF-PVA hydrogel without dring and re-swelling. It shows a stretchability of 91.4% and strength of 1.4 MPa.

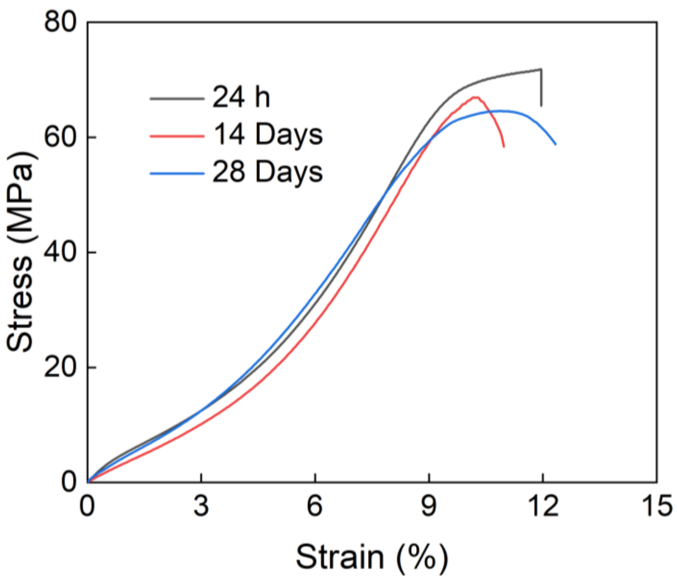

Fig. S10. Stability of ACH-80 in aqueous environment. The mechanical characteristics did not change over prolonged immersion in water.

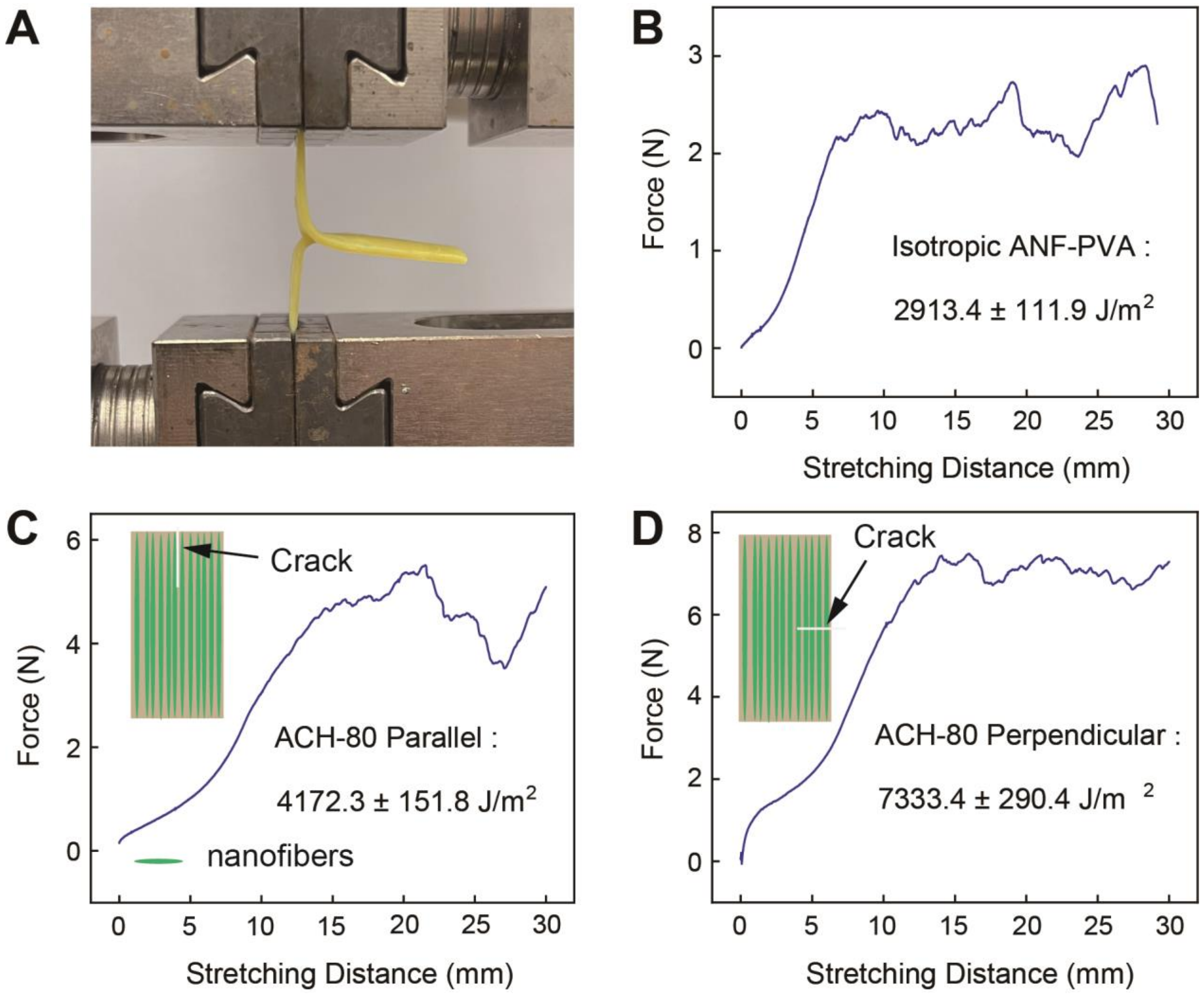

Fig. S11. Fracture energies of ACHs. (**A**) Photograph of a sample during a tearing test. (**B-D**) Force-extension curves and the corresponding fracture energies of isotropic ANF-PVA hydrogel (**B**), and ACH-80 measured in directions parallel (**C**) and perpendicular (**D**) to the fiber alignment.

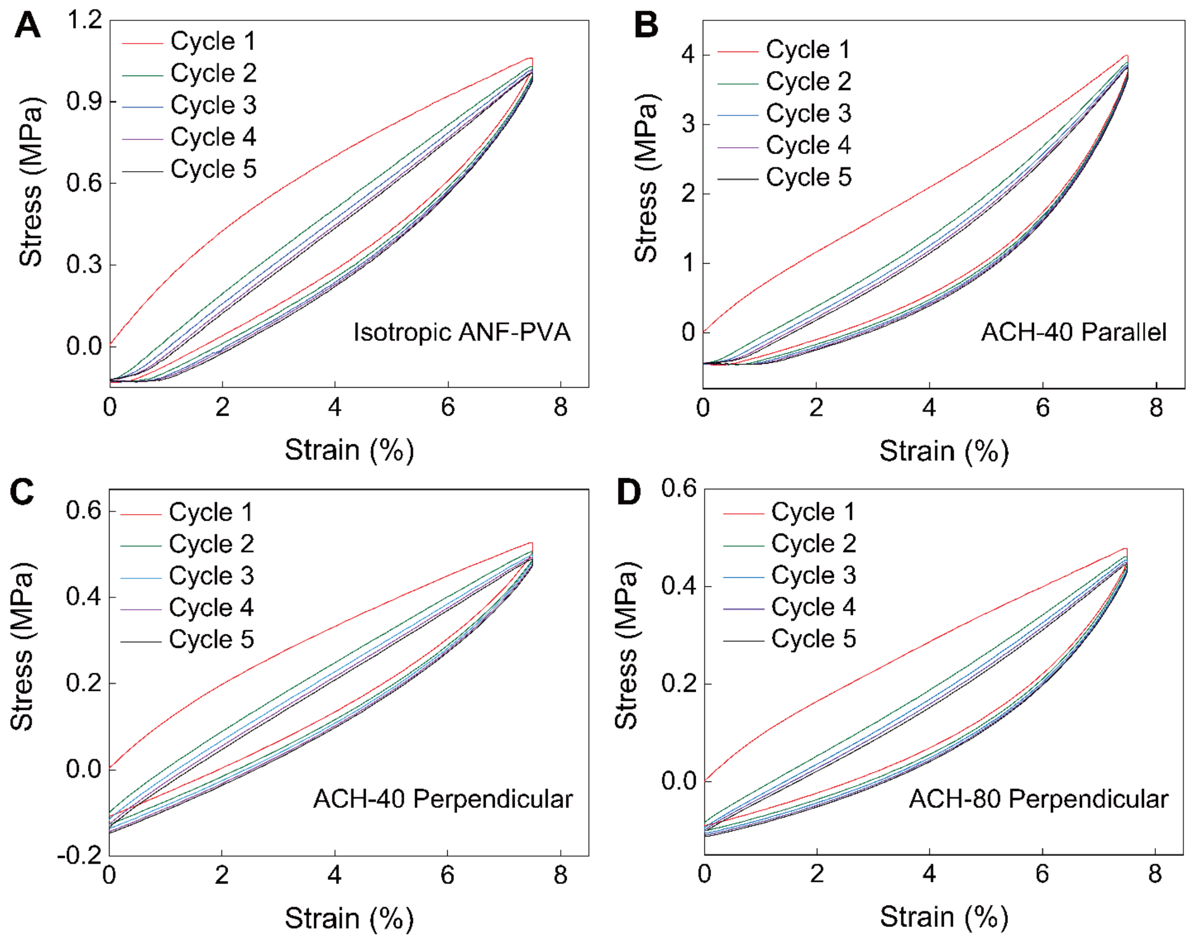

Fig. S12. Cyclic tensile tests on various samples with 7.5 % of maximum imposed strain. (**A**) Isotropic ANF-PVA hydrogel. (**B-C**) ACH-40 in the directions parallel (**B**) and perpendicular (**C**) to the fiber alignment. (**D**) ACH-80 in the direction perpendicular to the fiber alignment.

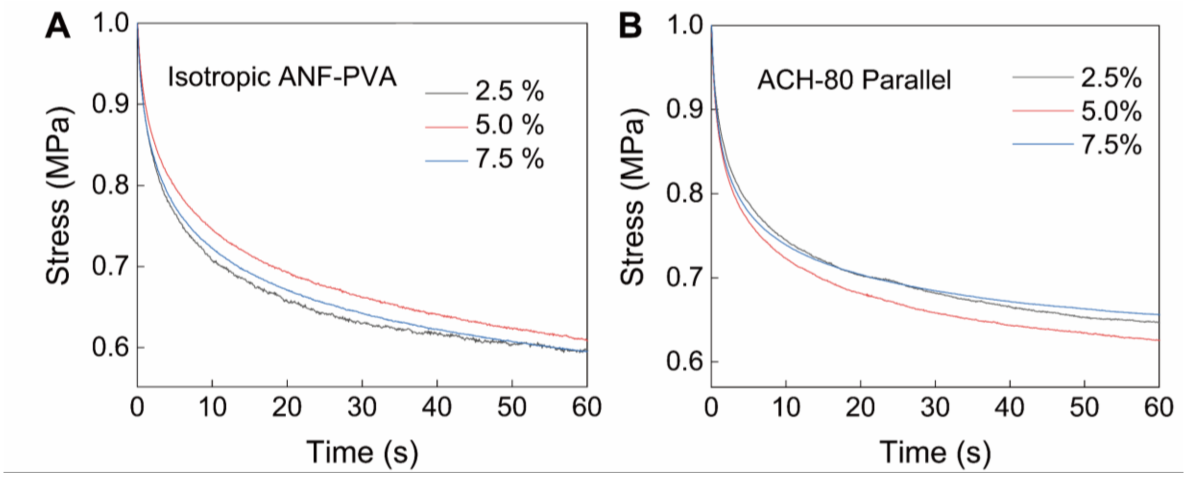

Fig. S13. Stress-relaxation behaviors of isotropic ANF-PVA (A) and ACH-80 (B) under various tensile strains imposed in the direction parallel to the fiber alignment. It shows a time constant on the order of ~10s, which is very similar to those of biological soft tissues (*36*).

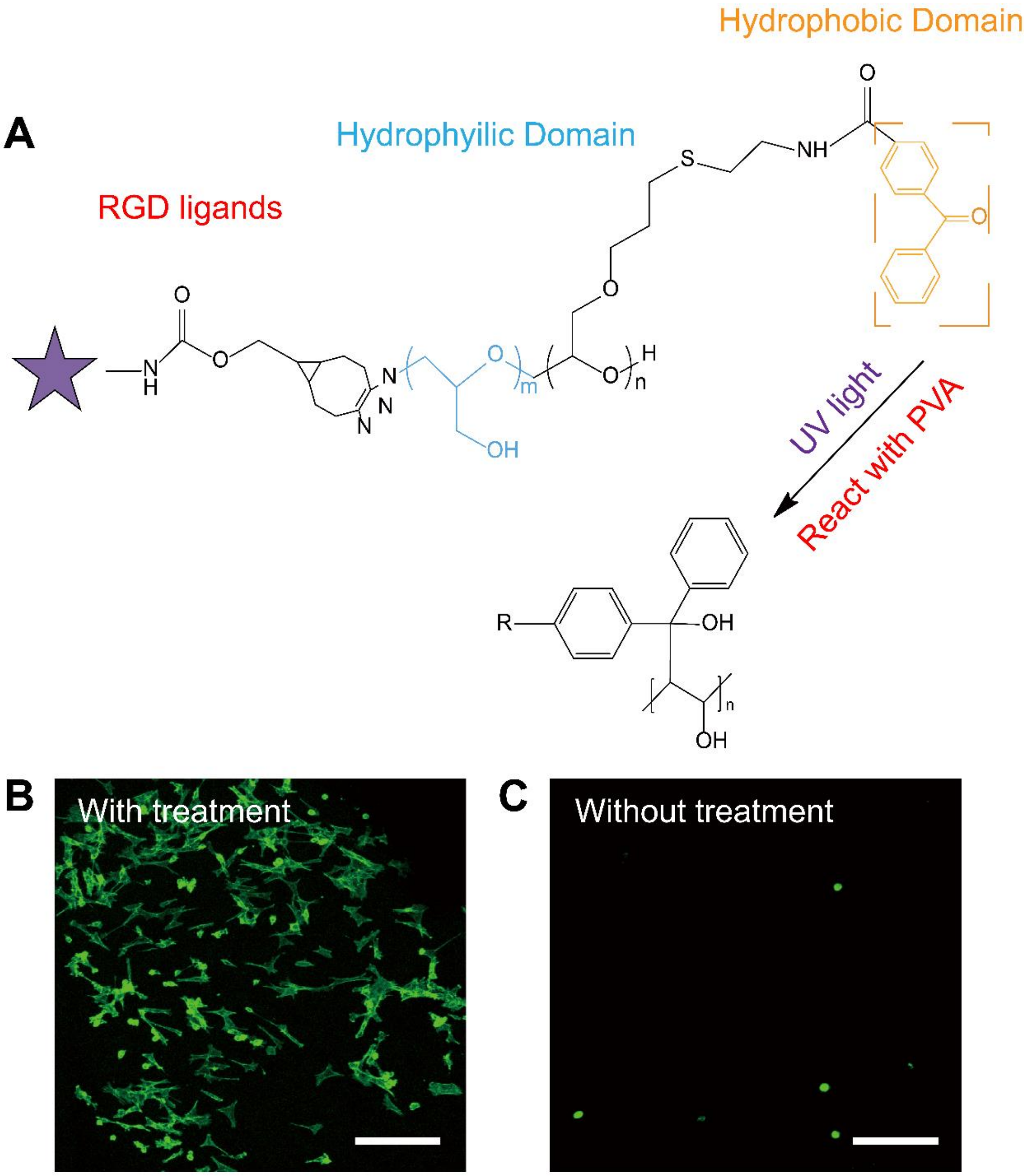

Fig. S14. Surface functionalization of ACH with integrin-binding RGD motif. (**A**) Chemical structures. (**B-C**) Confocal images of NIH3T3 fibroblasts cultured on ANF-PVA hydrogels with (**B**) and without (**C**) surface functionalization, indicating good attachment of cells on surface-functionalized samples. Scale bar: 100 µm.

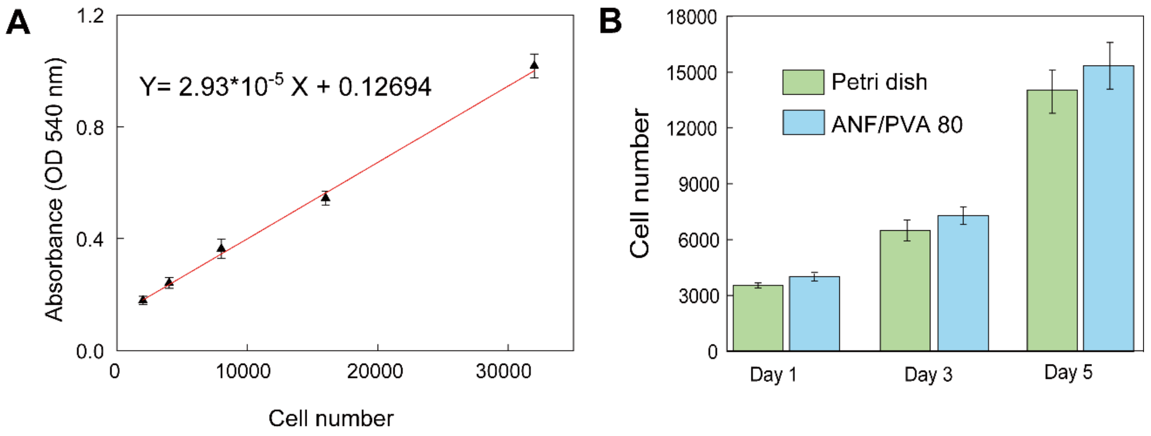

Fig. S15. Cell compatibility of biofunctionalized ACHs characterized with MTT assay. (**A**) Calibration curve of optical absorbance versus cell number. (**B**) Proliferation of NIH3T3 fibroblasts cultured on ACH-80 as compared with those cultured on a petri dish.

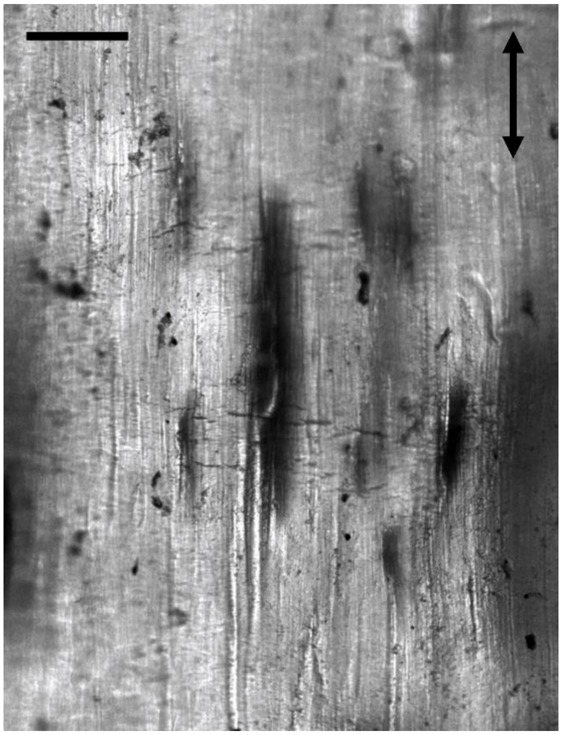

Fig. S16. Optical microscope image of ACH-80. It shows anisotropic surface topography, which may influence the morphology of attached cells via contact guidance. Scale bar: 100 µm.

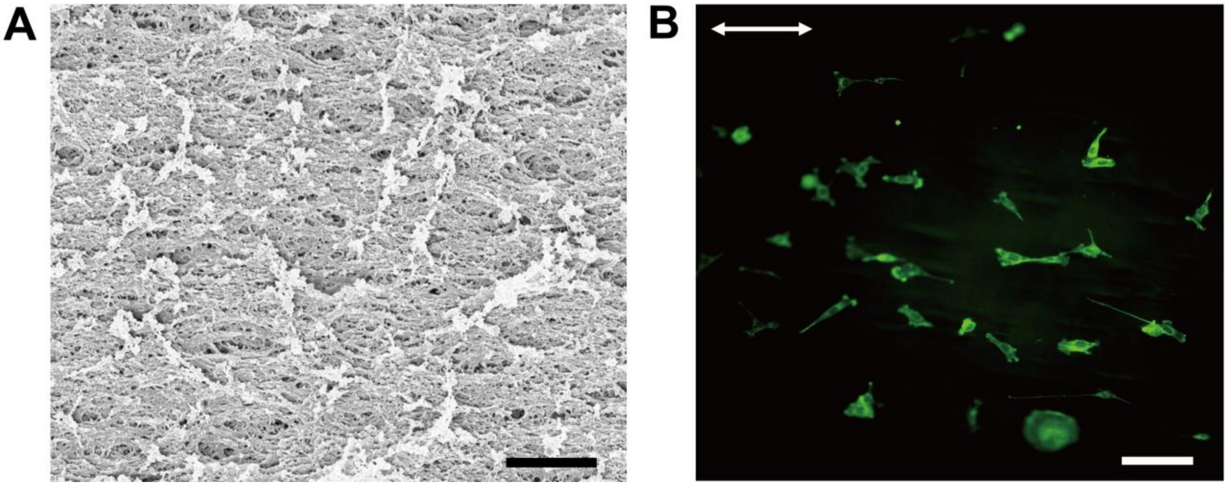

Fig. S17. Effect of plasma treatment on ACHs. (A) A SEM image showing the surface of ACH-80 after oxygen plasma treatment. Scale bar: 2 µm. (B) A fluorescence image of NIH3T3 firboblasts cultured on a ACH-80 treated with oxygen plasma etching for 2 h followed by re-swelling and biofunctionalization. The cells exhibit much less orientation as compared with those cultured on ACH-80 without etching. Scale bar: 100 µm.

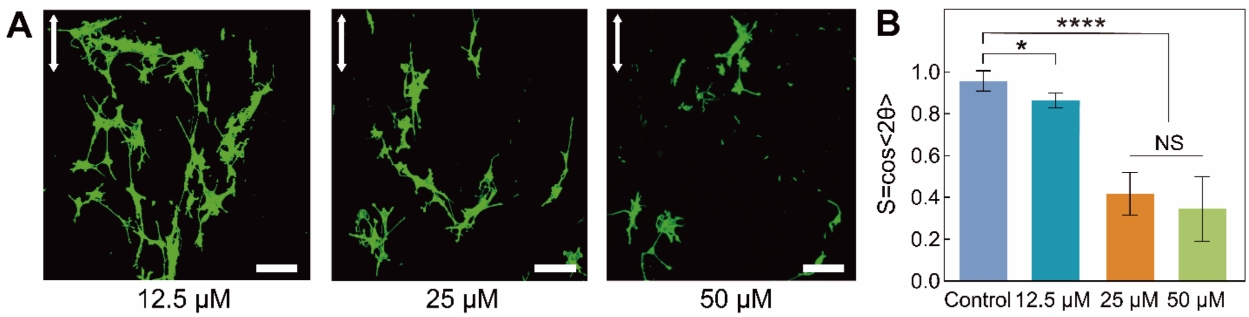

Fig. S18. Effects ROCK inhibitor Y-27623 on the morphology of NIH3T3 firboblasts cultured on ACH-80. (**A**) Fluorescent images of fibroblasts treated with various concentrations of Y-27632, showing the inhibition of cell orientation. Scale bar: 100 μm. **(B)** Quantitative analysis of cell orientational order parameter *S* in correlation with the concentration of Y-27632 (n=30, *P < 0.05, **P < 0.01, ***P < 0.001, ****P<0.0001). Orientation index (*S*) was determined by $S=\cos(2\theta)$.

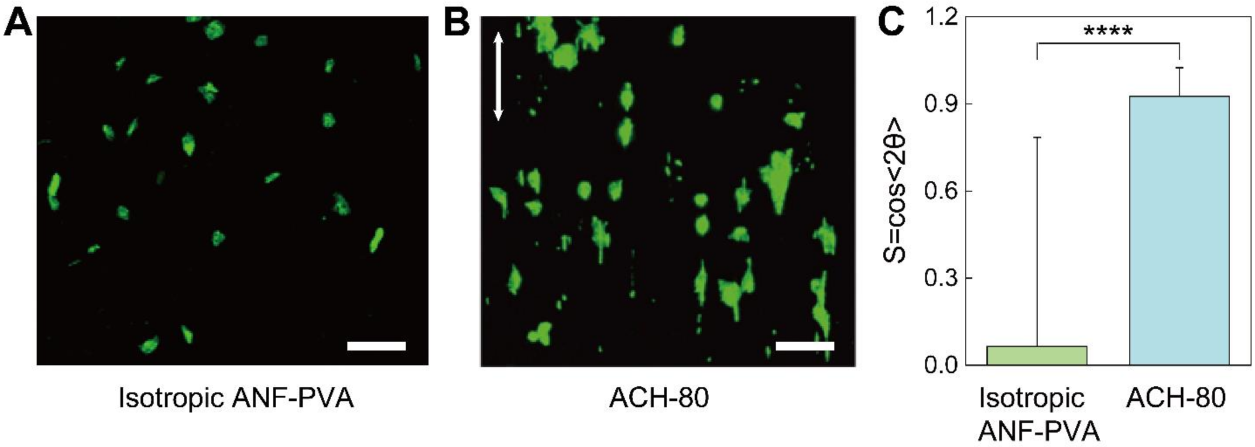

Fig. S19. Raw 264.7 macrophages cultured on various samples. (**A-B**) Florescent images of macrophages cultured on isotropic ANF-PVA hydrogel (**A**), and ACH-80 (**B**). Scale bar: 50 μm. (**C**) Statistics of macrophage orientational order parameter *S* (n=30).

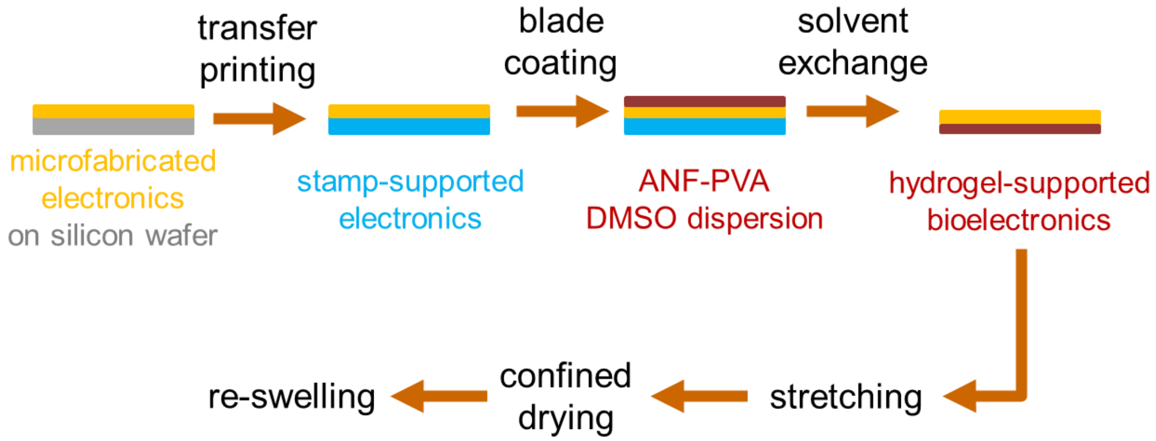

Fig. S20. Fabrication processes for hybrid ACHs with integrated bioelectronics. The devices can be microfabricated on a planar wafer and transfer-printed onto ANF-PVA hydrogels via solution processing.

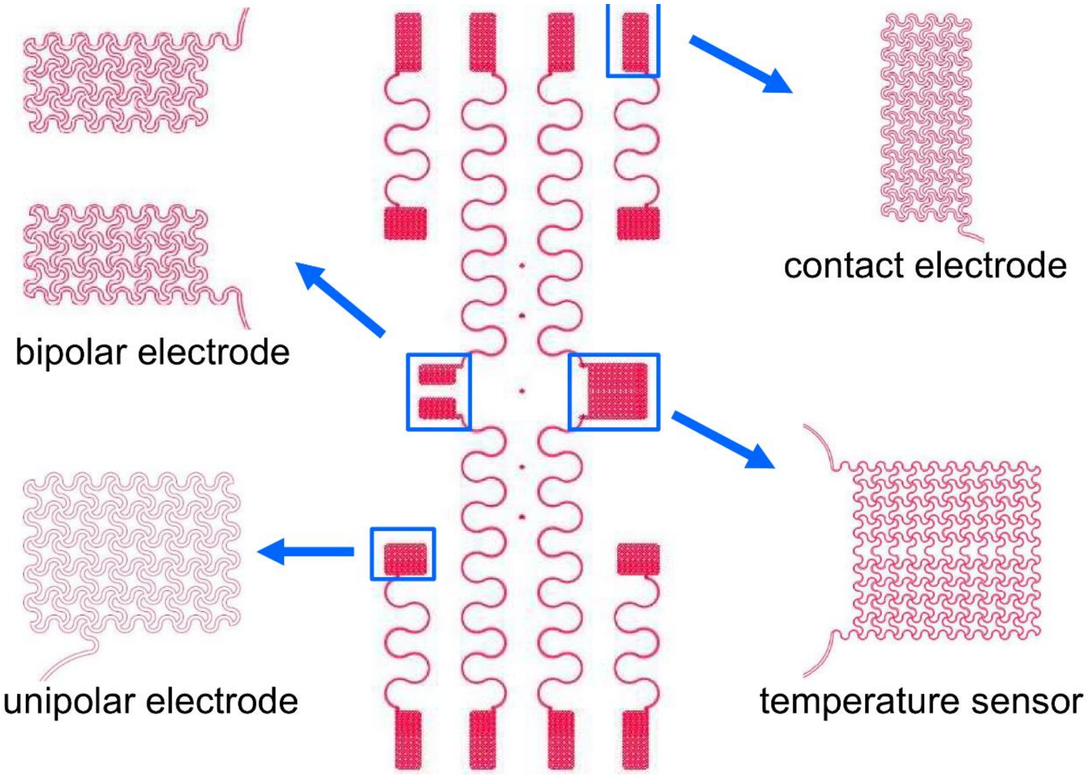

Fig. S21. The design of multifunctional sensors for the measurement of various physiological signals. The array includes bipolar electrodes, unipolar electrodes, temperature sensor and contact pads.

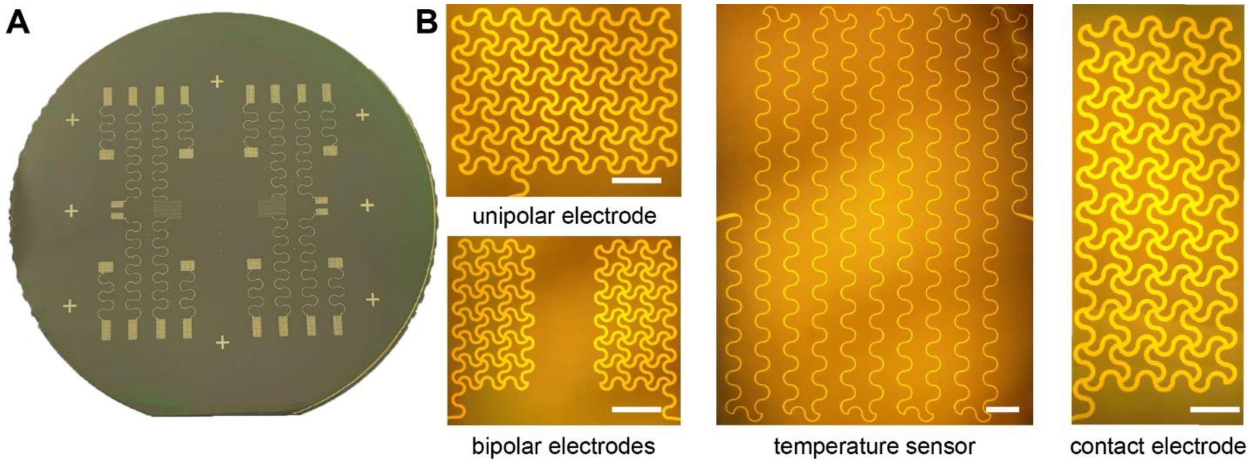

Fig. S22. Serpentine electronics for integration with ACHs. (**A**) An array of sensors fabricated on a 4-inch silicon wafer. (**B**) Optical microscope images showing the components of the device. (Scale bar: 1 mm.)

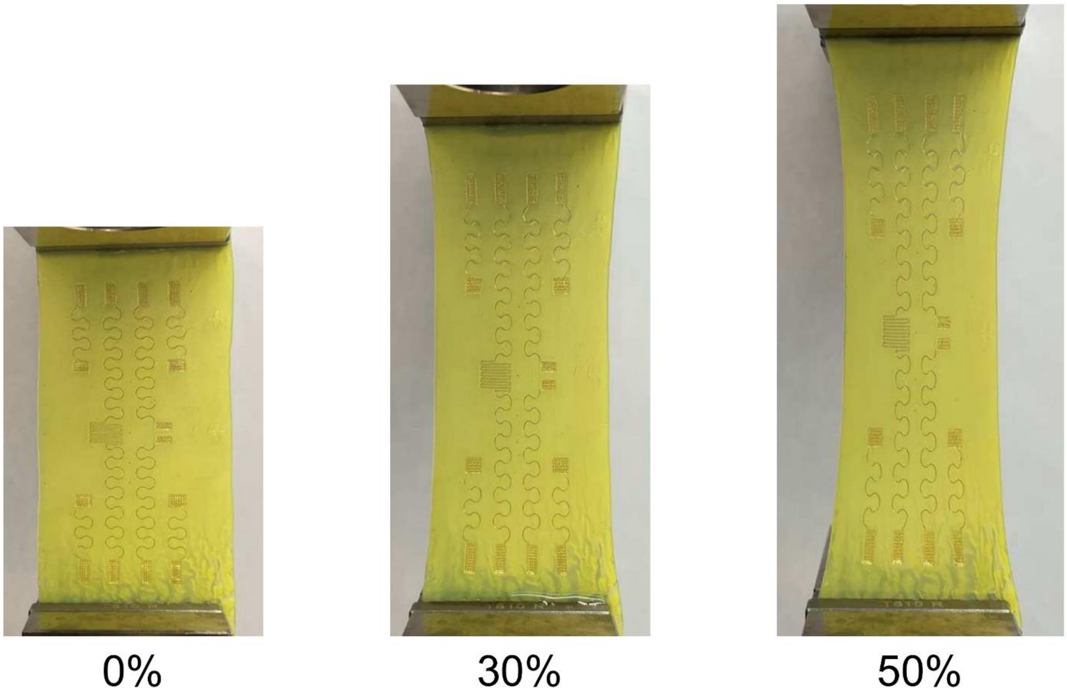

Fig. S23. Uniaxial stretching for the processing of an electronics-integrated ACH. There was no interfacial delamination or mechanical damage to the electronic components during the process, owing to the high stretchability of serpentine designs.

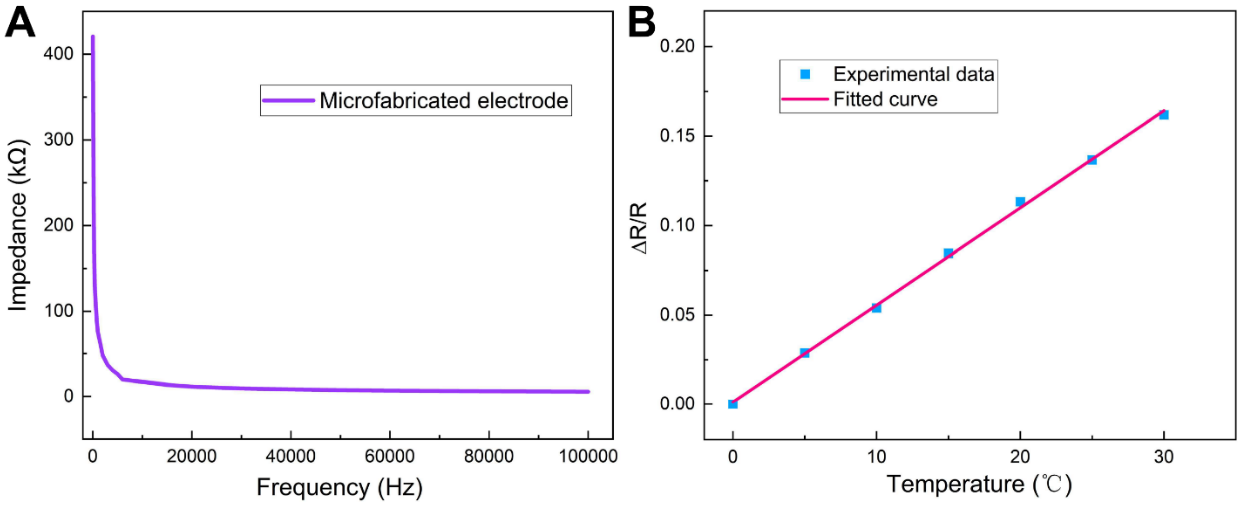

Fig. S24. Characteristics of the bioelectrodes and temperature sensors integrated on ACHs. (**A**) Electrical impedance between the microfabricated electrode and the skin as a function of frequency. (**B**) Change in resistance as a function of temperature, showing linear responses of the temperature sensor.

Table S1. Changes in liner dimensions of ACH samples during confined drying and reswelling. There was no major change in the length of the sample, indicating permanent alignment of the fibrillar network. The minor variation in the length after removing the stretching could be related to the entropic reconfiguration of PVA chains, leading to crimping of the fibers. In contrast, the significant changes in width and thickness indicate the variation of water content during the processes.

ACH-40	Dimensions	Before drying (confined) (mm)	After drying (confined) (mm)	Before re-welling (released) (mm)	After re-swelling (mm)
	Length	42.00 ± 0.02	42.00 ± 0.02	38.44 ± 1.31	39.47 ±1.62
	Width	2.61 ± 0.06	1.25 ± 0.05	1.32 ± 0.05	2.01 ± 0.04
	Thickness	1.12 ± 0.31	0.52 ± 0.08	0.55 ± 0.02	0.77 ± 0.08
ACH-60					
	Length	48.00 ± 0.01	48.00 ± 0.01	40.22 ± 0.85	40.25 ± 0.95
	Width	2.42 ± 0.22	1.18 ± 0.06	1.30 ± 0.03	1.99 ± 0.02
	Thickness	1.09 ± 0.12	0.50 ± 0.11	0.52 ± 0.06	0.73 ± 0.11
ACH-80					
	Length	54.00 ± 0.01	54.00 ± 0.01	44.02 ± 0.84	44.05 ± 1.14
	Width	2.29 ± 0.39	1.09 ± 0.09	1.27 ± 0.16	1.90 ± 0.01
	Thickness	0.95 ± 0.26	0.47 ± 0.17	0.50 ± 0.02	0.71 ± 0.01

Table S2. Mechanical properties of ACHs in comparison with other synthetic anisotropic hydrogels and natural biological tissues.

**Sample name**	Elastic modulus (MPa)	Ultimate Strength (MPa)	Reference
ACH-80	1114.0	72.1	This work
ACH-60	490.1	52.6	This work
FAS-PVA (freezing-assisted salting-out PVA)	2.5	23.5	(*13*)
MT-PVA (mechanically trained PVA)	0.2	5.2	(*8*)
DC-cellulose (dual crosslinked cellulose)	12.2	7.9	(*9*)
UD-PBDT-PAAM (uniaxially diffused poly (2,20-disulfonyl-4,40-benzidine terephthalamide) -polyacrylamide)	0.009	0.5	(*10*)
D-cellulose-PAM (delignified cellulose-polyacrylamide)	310	36	(*15*)
DCC-cellulose	342±31	53±0.5	(*19*)
DCC-alginate (drying in confined condition alginate)	367±54	20±2.2	(19)
MC-ligament (Medial collateral ligament)	332±58	39±5	(*37*)
AC-ligament (Anterior cruciate ligament)	447	46	(*4*)
PC-ligament (Posterior cruciate Ligament)	447	36	(*4*)
Achilles tendon	819±208	79±22	(*38*)
Patellar tendon	660±266	64.7±15.0	(*39*)

REFERENCES AND NOTES

1. C. F. Guimarães, L. Gasperini, A. P. Marques, R. L. Reis, The stiffness of living tissues and its implications for tissue engineering. *Nat. Rev. Mater.* **5**, 351–370 (2020).

2. H. Li, H. Liu, M. Sun, Y. Huang, L. Xu, 3D interfacing between soft electronic tools and complex biological tissues. *Adv. Mater.* **33**, 2004425 (2020).

3. A. Matson, N. Konow, S. Miller, P. P. Konow, T. J. Roberts, Tendon material properties vary and are interdependent among turkey hindlimb muscles. *J. Exp. Biol.* **215**, 3552–3558 (2012).

4. H.-J. Jung, M. B. Fisher, S. L. Y. Woo, Role of biomechanics in the understanding of normal, injured, and healing ligaments and tendons. *BMC Sports Sci. Med. Rehabil.* **1**, 9 (2009).

5. J. H.-C. Wang, Mechanobiology of tendon. *J. Biomech.* **39**, 1563–1582 (2006).

6. Y. Zhang, J. Yu, X. Wang, D. M. Durachko, S. Zhang, D. J. Cosgrove, Molecular insights into the complex mechanics of plant epidermal cell walls. *Science* **372**, 706–711 (2021).

7. S. Choi, Y. Choi, J. Kim, Anisotropic hybrid hydrogels with superior mechanical properties reminiscent of tendons or ligaments. *Adv. Funct. Mater.* **29**, 1904342 (2019).

8. S. Lin, J. Liu, X. Liu, X. Zhao, Muscle-like fatigue-resistant hydrogels by mechanical training. *Proc. Natl. Acad. Sci. U.S.A.* **116**, 10244–10249 (2019).

9. J. Zou, S. Wu, J. Chen, X. Lei, Q. Li, H. Yu, S. Tang, D. Ye, Highly efficient and environmentally friendly fabrication of robust, programmable, and biocompatible anisotropic, all-cellulose, wrinkle-patterned hydrogels for cell alignment. *Adv. Mater.* **31**, 1904762 (2019).

10. W. Yang, H. Furukawa, J. P. Gong, Highly extensible double-network gels with self-assembling anisotropic structure. *Adv. Mater.* **20**, 4499–4503 (2008).

11. J.-Y. Sun, X. Zhao, W. R. K. Illeperuma, O. Chaudhuri, K. H. Oh, D. J. Mooney, J. J. Vlassak, Z. Suo, Highly stretchable and tough hydrogels. *Nature* **489**, 133–136 (2012).

12. T. L. Sun, T. Kurokawa, S. Kuroda, A. Bin Ihsan, T. Akasaki, K. Sato, M. Haque, T. Nakajima, J. P. Gong, Physical hydrogels composed of polyampholytes demonstrate high toughness and viscoelasticity. *Nat. Mater.* **12**, 932–937 (2013).

13. M. Hua, S. Wu, Y. Ma, Y. Zhao, Z. Chen, I. Frenkel, J. Strzalka, H. Zhou, X. Zhu, X. He, Strong tough hydrogels via the synergy of freeze-casting and salting out. *Nature* **590**, 594–599 (2021).

14. Y. Z. Guo, T. Nakajima, M. T. I. Mredha, H. L. Guo, K. Cui, Y. Zheng, W. Cui, T. Kurokawa, J. P. Gong, Facile preparation of cellulose hydrogel with Achilles tendon-like super strength through aligning hierarchical fibrous structure. *Chem. Eng. J.* **428**, 132040 (2022).

15. W. Kong, C. Wang, C. Jia, Y. Kuang, G. Pastel, C. Chen, G. Chen, S. He, H. Huang, J. Zhang, Muscle-inspired highly anisotropic, strong, ion-conductive hydrogels. *Adv. Mater.* **30**, 1801934 (2018).

16. Y. J. No, S. Tarafder, B. Reischl, Y. Ramaswamy, C. Dunstan, O. Friedrich, C. H. Lee, H. Zreiqat, High-strength fiber-reinforced composite hydrogel scaffolds as biosynthetic tendon graft material. *ACS Biomater Sci. Eng.* **6**, 1887–1898 (2020).

17. Y. Huang, D. R. King, W. Cui, T. L. Sun, H. Guo, T. Kurokawa, H. R. Brown, C.-Y. Hui, J. P. Gong, Superior fracture resistance of fiber reinforced polyampholyte hydrogels achieved by extraordinarily large energy-dissipative process zones. *J. Mater. Chem. A* **7**, 13431–13440 (2019).

18. L. Xu, X. Zhao, C. Xu, N. A. Kotov, Water-rich biomimetic composites with abiotic self-organizing nanofiber network. *Adv. Mater.* **30**, 1703343 (2018).

19. M. T. I. Mredha, Y. Z. Guo, T. Nonoyama, T. Nakajima, T. Kurokawa, J. P. Gong, Hydrogels: A facile method to fabricate anisotropic hydrogels with perfectly aligned hierarchical fibrous structures. *Adv. Mater.* **30**, 1870060 (2018).

20. B. K. Hoffmeister, S. M. Handley, S. A. Wickline, J. G. Miller, Ultrasonic determination of the anisotropy of Young's modulus of fixed tendon and fixed myocardium. *J. Acoust. Soc. Am.* **100**, 3933–3940 (1996).

21. H. A. Lynch, W. Johannessen, J. P. Wu, A. Jawa, D. M. Elliott, Effect of fiber orientation and strain rate on the nonlinear uniaxial tensile material properties of tendon. *J. Biomech. Eng.* **125**, 726–731 (2003).

22. O. Chaudhuri, J. Cooper-White, P. A. Janmey, D. J. Mooney, V. B. Shenoy, Effects of extracellular matrix viscoelasticity on cellular behaviour. *Nature* **584**, 535–546 (2020).

23. L. Yu, Y. Hou, W. Xie, J. L. C. Camacho, C. Cheng, A. Holle, J. Young, B. Trappmann, W. Zhao, M. F. Melzig, Ligand diffusion enables force-independent cell adhesion via activating α5β1 integrin and initiating rac and RhoA signaling. *Adv. Mater.* **32**, 2002566 (2020).

24. A. Saez, M. Ghibaudo, A. Buguin, P. Silberzan, B. Ladoux, Rigidity-driven growth and migration of epithelial cells on microstructured anisotropic substrates. *Proc. Natl. Acad. Sci. U.S.A.* **104**, 8281–8286 (2007).

25. J. L. Charest, A. J. García, W. P. King, Myoblast alignment and differentiation on cell culture substrates with microscale topography and model chemistries. *Biomaterials* **28**, 2202–2210 (2007).

26. Z. Gong, S. E. Szczesny, S. R. Caliari, E. E. Charrier, O. Chaudhuri, X. Cao, Y. Lin, R. L. Mauck, P. A. Janmey, J. A. Burdick, Matching material and cellular timescales maximizes cell spreading on viscoelastic substrates. *Proc. Natl. Acad. Sci. U.S.A.* **115**, E2686–E2695 (2018).

27. C. H. Seo, K. Furukawa, K. Montagne, H. Jeong, T. Ushida, The effect of substrate microtopography on focal adhesion maturation and actin organization via the RhoA/ROCK pathway. *Biomaterials* **32**, 9568–9575 (2011).

28. Y. Zhu, H. Liang, X. Liu, J. Wu, C. Yang, T. M. Wong, K. Y. H. Kwan, K. M. C. Cheung, S. Wu, K. W. K. Yeung, Regulation of macrophage polarization through surface topography design to facilitate implant-to-bone osteointegration. *Sci. Adv.* **7**, eabf6654 (2021).

29. J. Hu, T. Wei, H. Zhao, M. Chen, Y. Tan, Z. Ji, Q. Jin, J. Shen, Y. Han, N. Yang, Mechanically active adhesive and immune regulative dressings for wound closure. *Matter* **4**, 2985–3000 (2021).

30. F. Y. McWhorter, T. Wang, P. Nguyen, T. Chung, W. F. Liu, Modulation of macrophage phenotype by cell shape. *Proc. Natl. Acad. Sci. U.S.A.* **110**, 17253–17258 (2013).

31. J. A. Fan, W.-H. Yeo, Y. Su, Y. Hattori, W. Lee, S.-Y. Jung, Y. Zhang, Z. Liu, H. Cheng, L. Falgout, M. Bajema, T. Coleman, D. Gregoire, R. J. Larsen, Y. Huang, J. A. Rogers, Fractal design concepts for stretchable electronics. *Nat. Commun.* **5**, 3266 (2014).

32. L. Xu, S. R. Gutbrod, A. P. Bonifas, Y. Su, M. S. Sulkin, N. Lu, H.-J. Chung, K.-I. Jang, Z. Liu, M. Ying, C. Lu, R. C. Webb, J.-S. Kim, J. I. Laughner, H. Cheng, Y. Liu, A. Ameen, J.-W. Jeong, G.-T. Kim, Y. Huang, I. R. Efimov, J. A. Rogers, 3D multifunctional integumentary membranes for spatiotemporal cardiac measurements and stimulation across the entire epicardium. *Nat. Commun.* **5**, 3329 (2014).

33. J. Koo, M. R. MacEwan, S.-K. Kang, S. M. Won, M. Stephen, P. Gamble, Z. Xie, Y. Yan, Y.-Y. Chen, J. Shin, Wireless bioresorbable electronic system enables sustained nonpharmacological neuroregenerative therapy. *Nat. Med.* **24**, 1830–1836 (2018).

34. L. Yu, Y. Hou, C. Cheng, C. Schlaich, P.-L. M. Noeske, Q. Wei, R. Haag, High-antifouling polymer brush coatings on nonpolar surfaces via adsorption-cross-linking strategy. *ACS Appl. Mater. Interfaces* **9**, 44281–44292 (2017).

35. C. Vergari, B. Ravary-Plumioen, D. Evrard, P. Laugier, D. Mitton, P. Pourcelot, N. Crevier-Denoix, Axial speed of sound is related to tendon's nonlinear elasticity. *J. Biomech.* **45**, 263–268 (2012).

36. P. P. Purslow, T. J. Wess, D. W. Hukins, Collagen orientation and molecular spacing during creep and stress-relaxation in soft connective tissues. *J. Exp. Biol.* **201**, 135–142 (1998).

37. K. M. Quapp, J. A. Weiss, Material characterization of human medial collateral ligament. *J. Biomech. Eng.* **120**, 757–763 (1998).

38. T. A. Wren, S. A. Yerby, G. S. Beaupré, D. R. Carter, Mechanical properties of the human achilles tendon. *Clin. Biomech.* **16**, 245–251 (2001).

39. G. A. Johnson, D. M. Tramaglini, R. E. Levine, K. Ohno, N. Y. Choi, S. L. Woo, Tensile and viscoelastic properties of human patellar tendon. *J. Orth. Res.* **12**, 796–803 (1994).
